# A potentially dangerous case of mistaken identity: giant asymptomatic composite phaeochromocytoma

**DOI:** 10.1530/EDM-25-0116

**Published:** 2025-12-23

**Authors:** Annabelle G Hayes, Morton G Burt

**Affiliations:** ^1^ The University of Adelaide, Adelaide, Australia; ^2^ Department of Endocrinology and Diabetes, Flinders Medical Centre, Bedford Park, Australia; ^3^ Flinders University, Adelaide, Australia

**Keywords:** phaeochromocytoma, composite, ganglioneuroma, adrenal tumour, subclinical phaeochromocytoma

## Abstract

**Summary:**

Small clinically silent phaeochromocytoma (PCC) can be identified in modern clinical practice as apparent adrenal incidentaloma or during screening of patients with familial tumour syndromes. Composite tumours comprising both PCC and a second tissue sharing embryological origin from the neural crest are rare, with fewer than 140 cases described in the literature. We report a 62-year-old woman with a 15 cm adrenal mass that was incidentally discovered on pulmonary imaging. A second 7 cm pelvic mass was also identified. The patient had no symptoms of catecholamine excess and normal blood pressure, even during a biopsy of the adrenal mass. Concordantly, urinary catecholamines were normal; however, urinary metanephrine and normetanephrine excretion were 23-fold and nine-fold the upper limit of normal, respectively. Surgical resection resulted in normalisation of metanephrines and normetanephrines. Histopathology showed a composite PCC/ganglioneuroma with discrete areas of both tumours within the same mass. Later resection of the pelvic mass revealed an unrelated ovarian teratoma. This case demonstrates a novel presentation of a composite PCC/ganglioneuroma and the presumptive role of catechol-O-methyltransferase in inactivating catecholamines within PCC, resulting in undetected growth of the tumour to a giant size. It highlights that metanephrines and normetanephrines are the preferred investigation for PCC.

**Learning points:**

- Clinically silent PCC are increasingly common but are typically small, with relatively low levels of metanephrines.
- Upregulation of COMT causes intratumoural inactivation of catecholamines and may facilitate asymptomatic growth of PCC to a giant size.
- Measurement of metanephrine and normetanephrine levels in plasma or urine is the preferred biochemical investigation for PCC.
- Composite PCC are rare but have a similar clinical presentation and management to other PCC.

## Background

Clinically silent phaeochromocytoma (PCC) are increasingly recognised but are typically small, with relatively low levels of metanephrines, as size correlates with biochemical activity and risk of metastasis (1). Alternatively, upregulation of catechol-O-methyltransferase (COMT), the enzyme that inactivates catecholamines to their metabolites in PCC, can result in clinical silence despite elevated metanephrine and normetanephrine levels. Composite PCC comprise tumoural tissue from both chromaffin cells and another cell line sharing embryological origin from the neural crest, including ganglioneuroma or neuroblastoma. While fewer than 140 cases have been described, they appear to demonstrate similar natural history and prognosis to other PCC but could be associated with higher rates of genetic mutations and distant disease recurrence (2, 3).

We report a novel presentation of a composite PCC/ganglioneuroma that highlights the likely role of COMT in inactivating catecholamines within PCC, allowing for undetected growth of the tumour to a giant size.

## Case presentation

A 62-year-old woman presented to the Emergency Department with left-sided pleuritic chest pain on a background of protein S deficiency and two prior deep vein thromboses. Computed tomography pulmonary angiography (CTPA) confirmed bilateral proximal pulmonary emboli with associated left lower lobe pulmonary infarction. She was recommenced on anticoagulation. The CTPA also obtained a partial view of a large right-sided abdominal mass, initially thought to be a cystic liver lesion.

**Figure 1:**
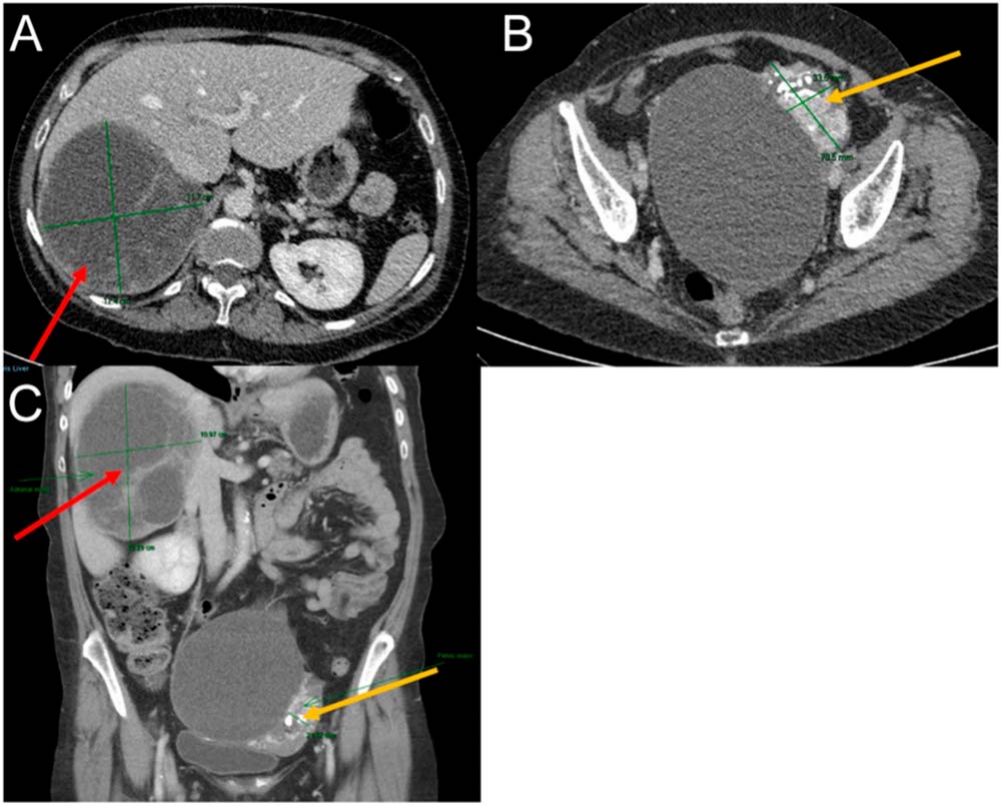
CT abdomen/pelvis in portal venous phase: (A) axial view of complex 15 cm right adrenal mass (red arrow), (B) axial view of heterogeneous 7 cm pelvic mass (yellow arrow), (C) coronal view of both masses (right adrenal mass (red arrow) and pelvic mass (yellow arrow)).

Further imaging with ultrasound and dedicated CT of the abdomen and pelvis revealed a large, circumscribed mass measuring 14 cm in maximal diameter that appeared to arise from the right adrenal gland (Fig. 1). Both ultrasound and CT described a multicystic lesion with intervening enhancing septa and areas of more intense small nodular enhancement. The solid portion of the tumour had a density of 16 Hounsfield units on non-contrast CT, and no macroscopic fat was seen. It broadly contacted but did not invade the inferior vena cava. A second 7 cm mass was identified in the pelvis with mixed solid, calcified, and cystic components.

Before referral to the Endocrinology Unit and contrary to our usual recommendations, a core biopsy of the adrenal mass was performed, which was not complicated by haemodynamic compromise. Histological examination was unable to yield a definitive diagnosis but was suggestive of a neuroblastic tumour.

Further history revealed no symptoms of a hormonally active PCC, including few headaches and an absence of palpitations, diaphoresis, or sense of doom. She did not take any regular medications. Besides her known coagulopathy, the patient had no significant medical history, nor any family history of endocrinopathy or endocrine tumours.

The patient’s blood pressure was 122/90 mmHg supine and 130/84 mmHg erect, with a heart rate of 72 beats per minute in sinus rhythm. There were no clinical features of neurofibromatosis. Clinical examination and echocardiogram did not demonstrate left ventricular hypertrophy or cardiomyopathy.

## Investigation

Urinary cortisol, adrenaline, and noradrenaline excretion over 24 h were within normal limits (Table 1). However, catecholamine metabolites were markedly elevated; in particular, urinary metanephrine excretion was 23-fold the upper limit of normal (Table 1). Similar results were found on repeat testing. Serum calcium, parathyroid hormone, and calcitonin were normal.

**Table 1:** Preoperative biochemical evaluation of adrenal medulla.

	6 months pre-operation	3 months pre-operation	Reference range
Plasma metanephrine			
pmol/L	2,749		<500
pg/mL	542		<98.6
Plasma normetanephrine			
pmol/L	5,537		<900
pg/mL	1,014		<165
Plasma 3-methoxy-tyramine			
pmol/L	148		<150
pg/mL	24.7		<25.1
24 h urinary adrenaline			
nmol/24 h	128	139	<150
μg/24 h	23.5	23.5	<27.5
24 h urinary noradrenaline			
nmol/24 h	435	361	<450
μg/24 h	73.6	61.0	<76.1
24 h urinary dopamine			
nmol/24 h	7,436	8,259	<3,500
μg/24 h	1,140	1,266	<536
24 h urinary metanephrine			
μmol/24 h	30.5	18.7	<1.3
μg/24 h	6.02	3.69	<0.26
24 h urinary normetanephrine			
μmol/24 h	28.2	47.8	<3.0
μg/24 h	5.16	8.64	<0.55
24 h urinary 3-methoxy-tyramine			
μmol/24 h	4.3	4.8	<2.7
μg/24 h	0.72	0.80	<0.45

**Figure 2:**
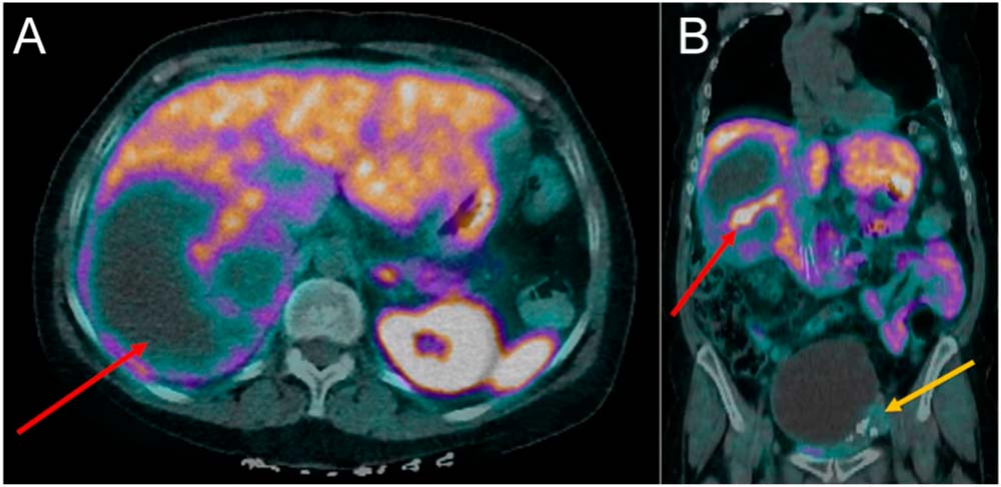
^68^Gallium-DOTATATE PET/CT. (A) Axial view of right adrenal mass (red arrow) demonstrating areas of low uptake due to a cystic, necrotic core. (B) Coronal view of both right adrenal mass (red arrow) and pelvic mass with low GaTate avidity (yellow arrow).

PET ^68^Gallium-DOTATATE scan demonstrated peripheral avidity of the adrenal mass, suggesting the adrenal mass was a neuroendocrine tumour with a cystic, necrotic core (Fig. 2). Low GaTate avidity of the ovarian mass suggested dissimilar tissue. There was no evidence of metastasis.

## Treatment

**Figure 3:**
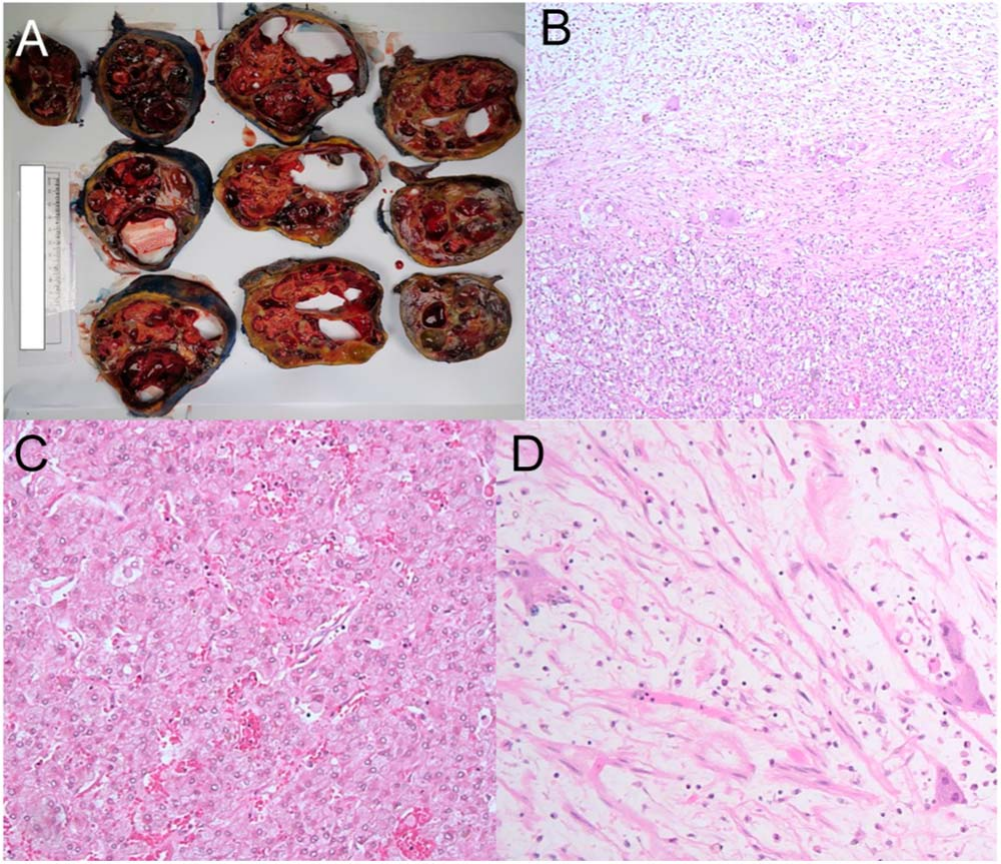
(A) Macroscopic examination of the tumour demonstrating a multicystic appearance, 4–64 mm in diameter, filled with clear watery and haemorrhagic fluid. (B) Loose fibrillary cells of ganglioneuroma superiorly, transitioning to phaeochromocytoma in the lower portion of the image. (C) High-powered film of phaeochromocytoma tissue with classic nested zellballen pattern. (D) High-powered film of ganglioneuroma tissue.

The patient was commenced on prazosin 0.5 mg twice daily, which was up-titrated to 2 mg twice daily before surgery. Laparoscopic-assisted adrenalectomy was performed after 6 months of therapeutic coagulation for her pulmonary emboli and proceeded without complication. Histological examination revealed a composite tumour with two distinct tumour types. One portion demonstrated the classic histological appearance of a PCC with a nested zellballen pattern of growth, while another portion was comprised of ganglioneuroma cells, comprising scattered ganglion cells within a fibrillary Schwannian stroma (Fig. 3). There was normal expression of SDHB immunohistochemistry and PHOX2B. The PASS score was 0, and the Ki-67 proliferative index was <1%.

## Outcome and follow-up

Plasma metanephrines and normetanephrines normalised 6 weeks post-operatively and have remained normal for 3 years. Subsequent surgical resection of the pelvic mass after adrenalectomy revealed a benign ovarian teratoma without a neuroendocrine component. The patient underwent genetic testing with next-generation sequencing (NGS), which did not find any variant of clinical significance in *FH, MAX, MEN1, NF1, RET, SDHA, SDHAF2, SDHB, SDHC, SDHD, TMEM127, or VHL.*

## Discussion

This case report describes a patient with a clinically silent giant composite PCC/ganglioneuroma discovered incidentally on CTPA. These characteristics are each very rare and are yet to be seen as a combined pathology in the published literature.

This mass was a composite tumour of PCC and ganglioneuroma tissue. Composite PCC are rare, with fewer than 140 cases described in the literature (2, 3, 4). They comprise PCC and a second tissue sharing embryological origin from the neural crest, including ganglioneuroma, ganglioneuroblastoma, neurobastoma, or schwannoma. Composite PCC typically present in the fifth decade at a median diameter of 3.8–5.7 cm (2, 3, 4, 5). Although data are limited, composite PCC are on average smaller than adrenal ganglioneuromas and comparable in size to PCC (3, 4). Most are diagnosed incidentally; although 85–95% of cases in series secrete catecholamines and/or their metabolites, only 25% of cases are identified due to symptoms of hormone excess (3, 4). Only 5% of cases presented due to mass effect, as compared to 11% of adrenal ganglioneuromas (4). In contrast to the cystic appearance of our case’s tumour, none of the 20 composite PCC in the series by Drages *et al.* had a cystic CT appearance (4). Median HU on non-contrast CT scans was 26.3 (range 19–32) (4). Based on limited follow-up data, prognosis appears similar to ordinary PCC, with metastatic disease recurrence reported, and lifelong surveillance is recommended (2, 3, 4, 5, 6).

Identification of clinically silent PCC is increasingly common in the current era of cascade genetic screening and high-resolution CT imaging, which account for 12 and 62% of PCC diagnoses, respectively (7). However, these subclinical lesions are typically small, with relatively low levels of metanephrines (7). Generally, tumour size correlates with biochemical activity of PCC and risk of metastasis (1, 7). In contrast, the case reported was a giant PCC with extremely high concentrations of metanephrine in plasma and urine but no evidence of excess adrenaline secretion. Moreover, the second tumour in the pelvis was unrelated; there were no metastases.

**Figure 4:**
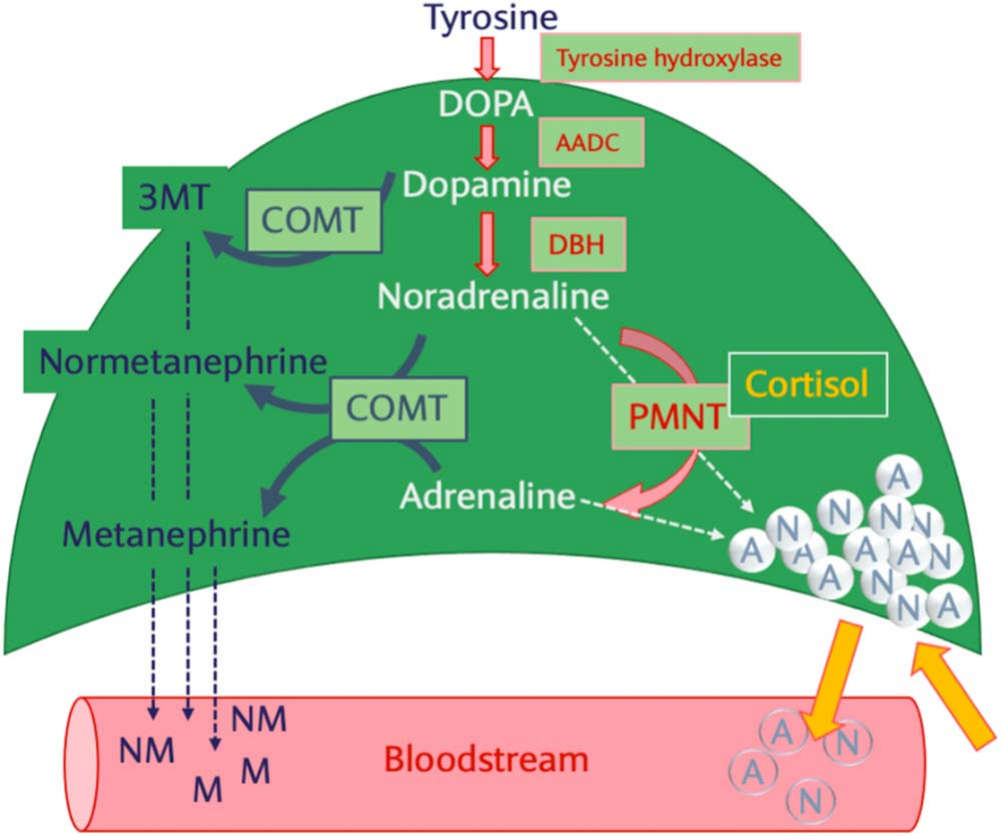
Catecholamine metabolism in the adrenal medulla. Aromatic L-amino acid decarboxylase (AADC), dopamine β-hydroxylase (DBH), phenylethanolamine N-methyltransferase (PNMT), catechol-O-methyltransferase (COMT).

An understanding of catecholamine metabolism by PCC is pivotal to explaining these biochemical findings. In the adrenal medulla, noradrenaline is produced from dopamine and converted to adrenaline by phenylethanolamine N-methyltransferase (PNMT) in the presence of cortisol from the nearby adrenal cortex (Fig. 4) (4). Catecholamines are stored in cytoplasmic vesicles and released upon stimulation or O-methylated by catechol-O-methyltransferase (COMT) to inactive metanephrine and normetanephrines (8). Given the absence of COMT in the sympathetic nerves, production of metanephrines is relatively specific to the chromaffin cells of the adrenal medulla (8). Furthermore, adrenergic PCC are well-differentiated tumours with mature catecholamine synthesis and feedback (8).

In this case, metanephrine levels were 23-fold normal, while adrenaline was well within the normal range. The likely explanation for this is markedly heightened catecholamine O-methylation by COMT. It has previously been reported that PCC can overexpress membrane-bound COMT; however, this is beyond what is expected (8). This likely allowed asymptomatic growth of the tumour to a very large size and explained the lack of haemodynamic sequelae after the adrenal biopsy. Nonetheless, we would not recommend nor endorse the practice of adrenal biopsy due to the risk of precipitation of catecholaminergic storm and haemodynamic collapse (9). Since this case, further hospital-wide education has been provided at our institution.

Metanephrines and normetanephrines are the preferred tests for investigation of PCC due to their stability in plasma, long half-life, and high specificity (1, 10, 11, 12). Seminal studies have demonstrated that plasma and urinary metanephrines have greater sensitivity and specificity than catecholamines to diagnose PCC (11). While this message is widely appreciated by endocrinologists, other physicians often still order catecholamines, as in this case presentation.

PCC can be subdivided based on their pattern of hormone secretion. Cluster 1 PCC lack expression of PNMT and therefore do not produce adrenaline (8, 10). They secrete noradrenaline and normetanephrine into the systemic circulation. In contrast, cluster 2 PCC typically have a more mature secretory pathway and express PNMT, resulting in production of adrenaline as well as noradrenaline. While they store high concentrations of both adrenaline and noradrenaline, they release very little into the periphery without provocation (1, 8). Consequently, diagnosis of cluster 2 tumours can be missed by measuring catecholamines, and quantification of plasma or urinary metanephrines is required, as demonstrated by our case.

As 30–35% of PCC arise due to an underlying germline mutation, it is recommended that all patients with PCC be considered for genetic testing (10, 12). The pattern of catecholamine secretion by a PCC provides insight into the potential genetic cause (1). Mutations in VHL, SDHB, and SDHD predominantly cause a cluster 1 phenotype, whereas mutations of RET, NF1, HRAS, or TMEM127 cause a cluster 2 pattern. Understanding expected biochemical phenotypes remains important in clinical practice, including interpreting identification of variants of uncertain significance (1, 10).

Of the initial 94 composite PCC cases published in the literature, a recognised genetic cause was found in 27%, comparable to that of classic PCC (2, 10). However, the recent series by Agarwal *et al.* found a higher rate of genetic mutations in composite vs classic PCC (47 vs 23%) across MEN2/4, SDHx, VHL, NF1, and EPAS1 (3). Neurofibromatosis type 1 appears overrepresented in composite tumours as compared to usual PCC, present in ∼19% of cases in whom genetic testing was performed, compared with 2–3% in other PCC cohorts (6). Genetic testing with NGS using a phaeochromocytoma panel did not identify a familial tumoural syndrome in our patient.

In summary, we describe an asymptomatic patient with a giant composite PCC. The tumour secreted normetanephrine and metanephrine but not active catecholamines. Presumptive upregulation of COMT could explain the clinical silence and biochemical findings. The case has a unique combination of massive size, rare histology, and marked metanephrine elevation relative to normal catecholamines but highlights the rationale for biochemical assessment of PCC in current guidelines (12).

## Declaration of interest

The authors declare that there is no conflict of interest that could be perceived as prejudicing the impartiality of the research reported by either author.

## Funding

No public or commercial funding has been received for this publication. The authors’ work was performed as employees of SA Health, the public health service of South Australia.

## Patient consent

Written informed consent was obtained directly from the patient for the publication of this case report.

## Author contribution statement

AH collected data and drafted the manuscript. MGB was involved in the diagnosis and management of the patient and revision of the manuscript. Both authors approve the final version of the manuscript.
